# Involvement of a novel *Pseudomonas protegens* strain associated with entomopathogenic nematode infective juveniles in insect pathogenesis

**DOI:** 10.1002/ps.7166

**Published:** 2022-09-19

**Authors:** Luca Ruiu, Maria Giovanna Marche, Maria Elena Mura, Eustachio Tarasco

**Affiliations:** ^1^ Dipartimento di Agraria University of Sassari Sassari Italy; ^2^ Dipartimento di Scienze del Suolo, della Pianta e degli Alimenti University of Bari ‘Aldo Moro’ Bari Italy

**Keywords:** nematode, pest management, EPN, symbiont, biological control

## Abstract

**BACKGROUND:**

The bioinsecticidal action of entomopathogenic nematodes (EPNs) typically relies on their symbiosis with core bacteria. However, recent studies highlighted the possible involvement of other noncore species. We have recently isolated a novel *Pseudomonas protegens* strain as a major agent of septicaemia in larvae of the wax moth, *Galleria mellonella*, infected with a soil‐dwelling *Steinernema feltiae* strain. The actual role of this bacterium in entomopathogenesis was investigated.

**RESULTS:**

The association of *P. protegens* with nematodes appeared to be robust, as supported by its direct and repeated isolation from both nematodes and insect larvae infected for several consecutive generations. The bacterium appeared to be well‐adapted to the insect haemocoel, being able to proliferate rapidly after the injection of even a small amount of living cells [100 colony forming units (CFU)] to a larva, causing its fast death. The bacterium also was able to act by ingestion against *G. mellonella* larvae [median lethal concentration (LC_50_) = 4.0 × 10^7^ CFU mL^–1^], albeit with a slower action, which supports the involvement of specific virulence factors (e.g. chitinases, Fit toxin) to overcome the intestinal barrier to the haemocoel. Varying levels of bacterial virulence were observed on diverse target Diptera and Lepidoptera.

**CONCLUSION:**

The soil‐dwelling bacterium *P. protegens* appears to have evolved its own potential as a stand‐alone entomopathogen, yet the establishment of an opportunistic association with entomoparasitic nematodes would represent a special competitive advantage. This finding contributes to a deeper understanding of the nematode–bacteria biocontrol agent complex and the deriving paradigm of their use as biological control agents. © 2022 The Authors. *Pest Management Science* published by John Wiley & Sons Ltd on behalf of Society of Chemical Industry.

## Graphical abstract

A novel strain of *Pseudomonas protegens* was found to be stably associated with the soil‐dwelling entomopathogenic nematode *Steinernema feltiae*. This noncore bacterium can act as a stand‐alone bioinsecticide or may take advantage of the vector nematode to reach the insect haemocoeal where it can proliferate.

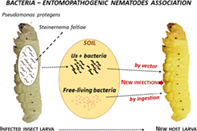

## 1 INTRODUCTION

The bioinsecticidal action of entomopathogenic nematodes (EPNs) typically relies on the symbiosis of these invertebrates with resident bacteria which they host in their gut.^1^ Once nematodes have actively entered the host via its natural openings (i.e. mouth, spiracles, anus), bacteria are released in the haemocoel where they can replicate, producing metabolites and substances that suppress the insect immune‐response capacity and create a suitable environment for nematode reproduction.^2^ This symbiosis model has been the subject of numerous studies aimed at understanding the interaction established between the host and the nematode that carries bacteria, which has led to the identification of so‐called bacterial core species, such as those belonging to the genera *Photorhabdus* and *Xenorhabdus*, normally associated with nematodes in the genera *Heterorhabditis* and *Steinernema*, respectively.^3^

However, other bacterial species, especially in the phylum Proteobacteria, occasionally have been isolated from EPN bodies.^4^, ^5^ Consistently, the new generation of 3rd stage infective juvenile nematodes (IJs), emerging from the body of a dead host, after incorporating core bacteria at the end of the infection cycle, have a life phase in the soil where they can come into contact with a variety of other microorganisms, which may cause an increase in their bacterial community diversity, involving noncore species that they might host. However, given its occasional nature, the possible implication of noncore bacteria in the nematode–host interaction previously has not been considered to have significant importance in pathogenicity.^5^

In a more recent study on the *Xenorhabdus namatophila*–*Steinernema carpocapsae* symbiotic interaction, alongside the well‐established hypothesis of monoxenic symbiosis, an additional pathogenic role was associated also with several proteobacterial species within the so‐called frequently associated microbiota (FAM).^6^ Accordingly, *S. carpocapsae* was found to be frequently associated with *Alcaligenes*, *Stenotrophomonas*, *Pseudomonas* and the Rhizobiaceae family, among which a possible involvement of *P*. *protegens* and *P*. *chlororaphis* in pathogenicity was highlighted. *Pseudomonas protegens* is a soil‐dwelling emerging species that has developed the ability to both establish beneficial interactions with plants and act as an insect pathogen.^7^ Interestingly, this bacterium harbours several insecticidal virulence factors found also in other entomopathogens. Amongst these, the fluorescent insecticidal toxin (Fit) complex showing significant homology to the makes caterpillar floppy (Mcf) toxin produced by the EPN core bacterial symbionts, *Photorhabdus* and *Xenorhabdus*, which supports a common evolutionary process for all of these bacterial species.^8^ However, the ecological mechanisms leading to such evolutionary events still need to be understood.

During a screening of a recent EPN collection, we have isolated a new *P. protegens* strain (CO1) from *Galleria mellonella* L. (Lepidoptera: Pyralidae) larvae infected by a *Steinernema feltiae* strain.^9^, ^10^ Because this bacterium was the dominant species in the haemolymph of septicaemic larvae, we hypothesized its involvement in the pathogenic process, as opposed to the better‐known role of *Xenorhabdus* core symbionts. Different experiments were conducted to investigate the involvement of *P. protegens* in pathogenicity and the robustness of its relationship with the nematode. The potential of *P. protegens* as a stand‐alone entomopathogen also was evaluated.

## 2 MATERIALS AND METHODS

### 2.1 Bacterial detection in nematodes

A first set of experiments was conducted to isolate *P. protegens* from nematodes reared *in vivo* for several generations of infection. For this purpose, 3rd instar *G. mellonella* larvae were infected with *S. feltiae* IJs and incubated on wet filter papers inside Petri dishes at 25° C to allow nematodes to complete their infection cycle reproducing inside the insect body.^11^ Next‐generation IJs emerging from dead larvae then were collected and used to infect new *G. mellonella* larvae, after saving nematode samples for analysis. At the same time, a sample of haemolymph was collected from septicaemic larvae for analysis. This infection process was repeated for several generations throughout a two‐year period. Haemolymph samples were diluted and used to inoculate Luria‐Bertani (LB) agar plates for *P. protegens* isolation. Colonies of this species could be identified on the Petri dishes by observation of their typical morphological features (i.e. pinkish colour) and occasionally by *16S* rDNA gene amplification and sequencing.^9^ Different generations of *S. feltiae* IJs emerging from dead larvae were surface‐sterilized by washing in 1% sodium hypochlorite, before being homogenized with a pestle in phosphate‐buffered saline (PBS). The homogenate was used to inoculate LB agar plates, after serial dilution, and for total DNA extraction using the Wizard® Genomic DNA Purification Kit (Promega, Madison, WI, USA) in compliance with the manufacturer's instructions. After being quantified by a NanoDrop ND‐1000 Spectrophotemeter (Thermo Fisher Scientific, Waltham, MA, USA), DNA samples were used as templates in polymerase chain reaction (PCR) employing Taq DNA Polymerase according to manufacturer's protocol (Promega), in a total volume of 25 μL, at the following conditions: 95 °C for 5 min, 30 cycles at 95 °C for 30 s, 58 °C for 45 s and 72 °C for 30 s, followed by 72 °C for 10 min.

Multiple primer pairs were designed to specifically target *P. protegens 16S* rDNA and cytotoxin *fitD* genes (Table 1). PCR products were visualized by electrophoresis on agarose gel (1%) using SYBR® Safe DNA stain (Life Technologies Europe BV, Bleiswijk, the Netherlands).

**Table 1:** Sequences of primer pairs used for PCR and qPCR analyses

Gene	Analysis	Primer sequence		Amplicon size (bp)
		Sense 5′‐ 3′	Antisense 5′‐ 3′	
*16S* rDNA	PCR	GCGAGCGGCGGACGGGTGAGTAAT	TTCCACCACCCTCTACCATACTCTAGC	500
*16S* rDNA	PCR	GCGAGCGGCGGACGGGTGAGTAAT	TGTACAAGGCCCGGGAACGTATTCACCG	1000
*fitD*	PCR	CGCCAACACCGAGCCACAG CCGGAGG	GGTAGGCCTTGTCCAGGGTGTCGAAGTAA	700
*16S* rDNA	qPCR	TTCCACCACCCTCTACCATACTCTAGC	TGGGAGGAAGGGCAGTTACCTAATACGTGA	‐

With the purpose of evaluating the robustness of the *S. feltiae–P. protegens* association, the same infection experiment was conducted on a different insect host, 3rd instar larvae of *Musca domestica* L. New‐generation nematodes emerging from dead larvae previously inoculated with *S. feltiae* IJs were analyzed for the presence of *P. protegens* as described previously.

### 2.2 Nematode‐carried bacteria reproduction in the insect body

In order to evaluate the actual involvement of *P. protegens* in the entomopathogenic process, the overtime dynamic of its load in the haemolymph of *G. mellonella* 3rd instar larvae was monitored by both bacterial colony counts (CFU) and quantitative (q)PCR targeting the *16S* rDNA gene with a specifically designed primer pair showing high affinity for *P. protegens* with respect to other nematode‐associated bacteria including *Xenorhabdus* spp.,^12^ as shown in Table 1. To this end, haemolymph was taken from larvae at different time intervals post‐inoculation with IJ (0, 12, 24, 36, 48, 60 and 72 h). The experimental design involved three pools of ten larvae for each time interval, and an aliquot (10 μL) from each larva was mixed with that of other larvae in the pool. For colony counts, 10 μL from each pool were serially diluted in PBS (pH 7.4) before being inoculated into LB agar plates and incubated at 30 °C for CFU assessment. Recognition of *P. protegens* colonies, which represented the vast majority of the culturable bacteria present, was carried out easily as a consequence of their typical morphology and pinkish colour. For qPCR analyses, after DNA extraction from haemolymph samples, Power SYBR® Green PCR Master Mix was used on an Applied Biosystems 7900HT Fast Real‐Time PCR System according to the manufacturer's instructions at the following conditions: denaturation at 95 °C for 10 min, followed by 40 cycles of 95 °C for 15 s, annealing at 60 °C for 1 min and extension at 60 °C for 1 min. These analyses were conducted in technical triplicates for each biological sample and detected gene abundance at different time intervals after infection (as a relative figure compared with time zero) was determined according to Livak and Schmittgen.^13^

### 2.3 Evaluation of *P. protegens* stand‐alone entomopathogenic properties

The entomopathogenic potential of the *P. protegens* strain isolated from nematodes was evaluated by bioassays conducted on *G. mellonella* 3rd instar larvae exposed either orally to or by injection of different dosages of bacterial cells obtained by centrifugation of a 72 h LB broth culture at 30 °C. For this purpose, starved larvae were treated by (i) intrahaemocoelic injection, using a Hamilton syringe mounted in an automatic syringe pump injecting 10 μL per larva; (ii) force‐feeding, employing the same syringe pump system injecting through the needle 10 μL in the mouth of each larva. In this way larvae were exposed to the same dose of bacterial CFU by ingestion or injection in a range between 10^2^ and 10^8^ CFU per larva. Control larvae were treated with an equivalent volume of 0.9% NaCl solution. The experimental design involved four replicates of ten larvae for each treatment. After being treated, larvae belonging to the same replicate were maintained in a Petri dish inside an incubator at 25 °C and inspected after 48 h to assess mortality. This experiment was repeated three times with different cohorts of larvae and bacterial cultures.

### 2.4 Target range evaluation

The insecticidal potential of this *P. protegens* strain was evaluated on a variety of insect species in the Lepidopteran and Dipteran orders. These included: Lepidoptera: corn earworm *Helicoverpa armigera* (Hübner) (Noctuidae) and gypsy moth *Lymantria dispar* L. (Erebidae): Diptera: *M. domestica* L. (Muscidae) and Mediterranean fruit fly *Ceratitis capitata* Wied. (Tephritidae). All bioassays were conducted in an incubator at 25 °C.

#### 2.4.1 Bioassays with Lepidoptera

Larvae of *H. armigera* were collected from tomato fields in Arborea (Sardinia, Italy) during spring, and maintained in the laboratory on fresh tomato leaves until being used in bioassays. Larvae of *L. dispar* hatching from egg masses collected in the forest of Sardinia (Italy) at the end of winter were maintained on wheat germ artificial diet^14^ until being used in bioassays.

For injection bioassays, a dose of 10^4^ CFU per larva was inoculated on 3rd instar larvae of the two lepidopteran species in the same way as previously described for *G. mellonella*.

For ingestion assays, four groups of 2nd instar larvae of each species were maintained in Petri dishes (10‐cm diameter) and fed *ad libitum* fresh leaves (tomato leaves for *H. armigera* and cork oak leaves for *L. dispar*), previously sprayed with the bacterial suspension at 10^9^ CFU mL^–1^ (treated) or just water (control).^15^ These experiments had four replicates and mortality was assessed daily for 72 h.

#### 2.4.2 Bioassays with Diptera

Larvae of *M. domestica* and *C. capitata* used in bioassays were provided by the insect rearing facility of the Department of Agricultural Sciences of the University of Sassari (Italy).^16^

Four groups of ten 3rd instar larvae of each species, after being injected a dose of 10^4^ CFU per larva, were maintained in petri dishes to assess mortality. For ingestion bioassays, 1st instar larvae in groups of ten individuals inside petri dishes, were reared on an artificial diet (2 g) incorporating *P. protegens* CO1 at a concentration of 10^9^ CFU g^–1^ or left untreated (control). The diet consisted of wheat bran (34%), milk powder (1%) and water (65%) (w/w) for *M. domestica*,^17^ and wheat bran (24.9%), saccharose (16.0%), yeast powder (8.0%), citric acid (0.6%) and water (50.5%) (w/w) for *C. capitata*.^18^ These experiments had four replicates and mortality was assessed daily for 72 h.

### 2.5 Statistical analysis

Statistical analyses were conducted using R software v4.0.4.^19^

The relationship between *P. protegens* abundance in larval haemolymph and time was determined by one‐way ANOVA followed by least significant difference (LSD) test for bacterial CFU counts and by linear regression analysis for real‐time PCR analyses.

Data on mortality after ingestion or injection of different bacterial doses were analyzed by two‐way ANOVA (factors: treatment, dose) followed by an LSD test to separate treatment means.

Dose–mortality data after feeding or injection treatments with increasing bacterial doses were subjected to probit analysis^20^ to determine median lethal concentration (LC_50_) within 95% confidence inervals (CI). In experiments with different insect targets, data on mortality after treatment by ingestion or injection were analysed using Student's *t*‐tests for mean comparison between treated and control groups.

## 3 RESULTS

### 3.1 Bacterial detection in nematodes

**Figure 1:**
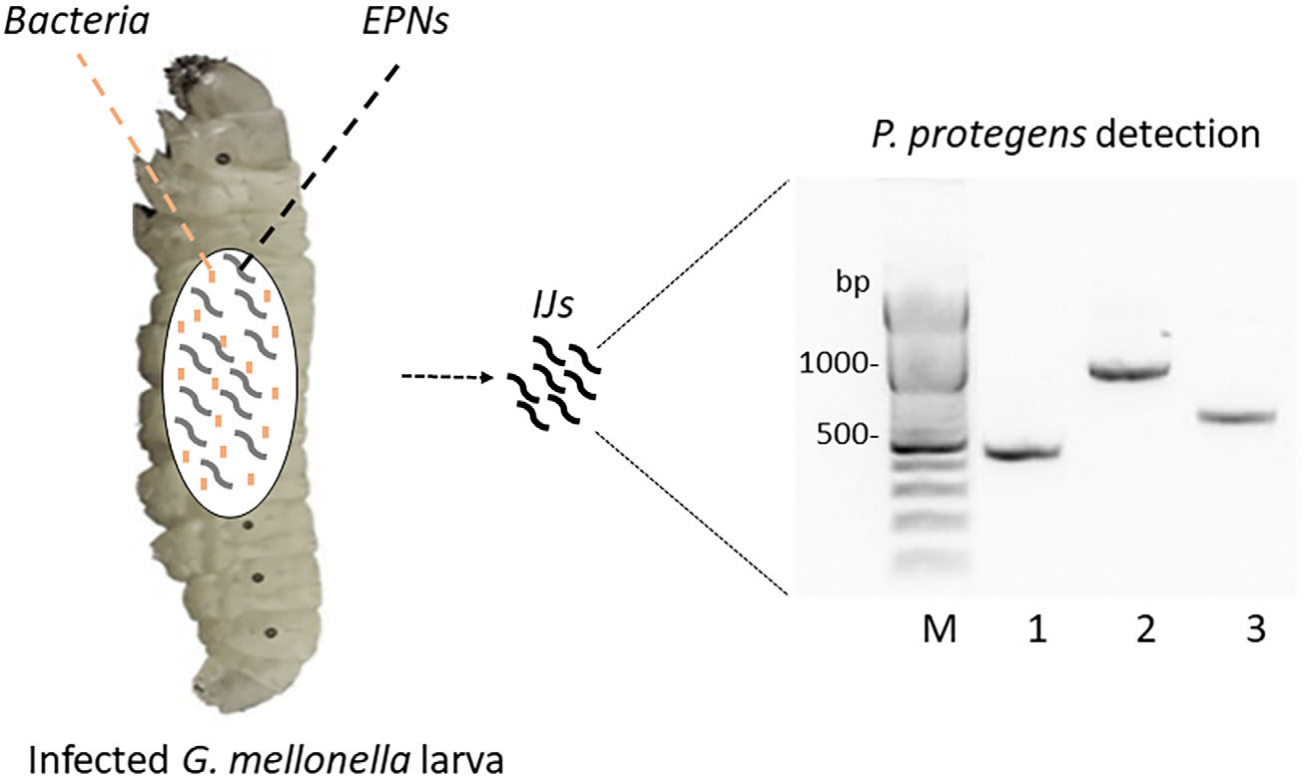
Representative agarose gel showing *P. protegens* detection in nematode juveniles with primer pairs targeting *16S* rDNA (amplicon size 500 and 1000 bp; lanes 1 and 2, respectively) and *fitD* gene (amplicon size 700 bp; Lane 3). M = 1 kb DNA ladder (Thermo Fisher Scientific) with the brightest band corresponding to 500 bp.

Based on microbial culture and PCR assays targeting *16S* rDNA and *fitD* genes, *P. protegens* was successfully detected in all samples of IJs from different generations of infection on *G. mellonella* larvae (Fig. 1). Likewise, these genes were found in nematodes emerging from infected larvae of *M. domestica*.

### 3.2 Bacterial load in IJ‐infected larvae

**Figure 2:**
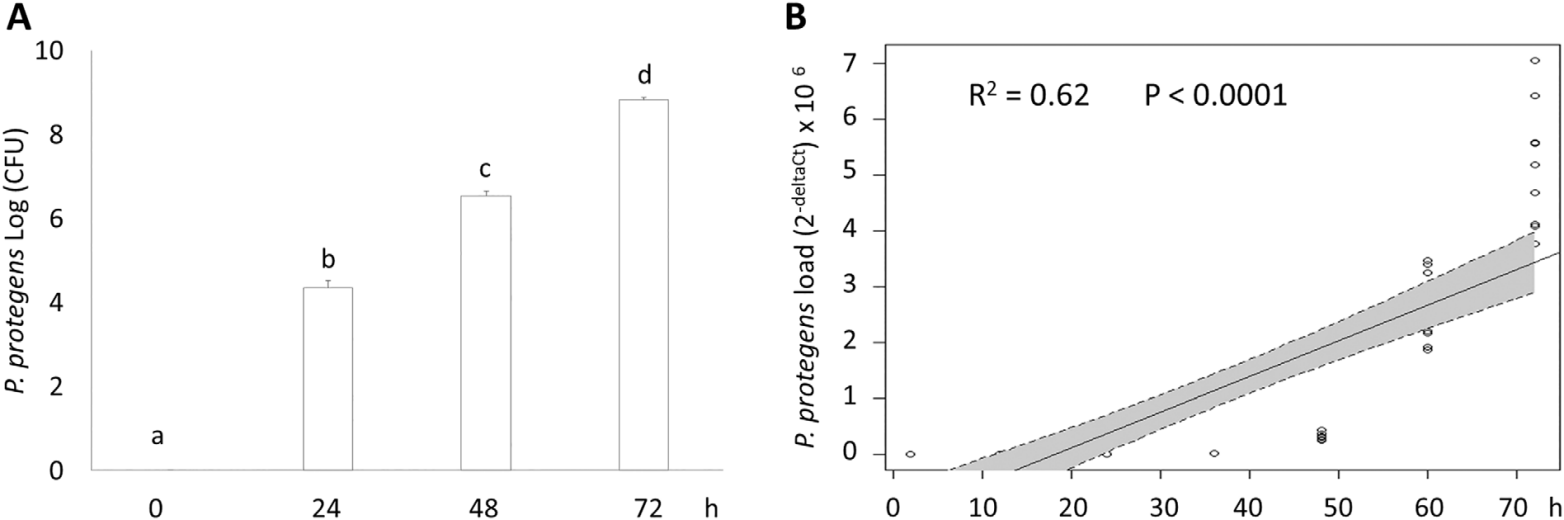
*Pseudomonas protegens* overtime load in the haemolypmph of *G. mellonella* larvae after *Steinernema* IJ infection. (A) Bacterial CFU at progressive time intervals (different letters above bars indicate significantly different means; ANOVA followed by LSD test, *P* < 0.001). (B) Linear regression plot with 95% CI (shaded areas) showing the predicted relationship between bacterial load determined by qPCR targeting the *16S* rDNA gene (expressed as 2^‐deltaCt^) and time.

The progressive abundance of *P. protegens* in the haemolymph of *G. mellonella* larvae infected with *Steinernema* infective juveniles was determined successfully by counting the number of bacterial CFU (*F*_3,35_ = 10 448.87, *P* < 0.0001) and by qPCR reactions (adjusted *R* ^2^ = 0.62, *F*_1,61_ = 101.8, *P* < 0.0001), and a significant overtime increase in bacterial load in infected larvae was observed, as shown in Fig. 2.

### 3.3 Stand‐alone virulence of *P. protegens*

**Figure 3:**
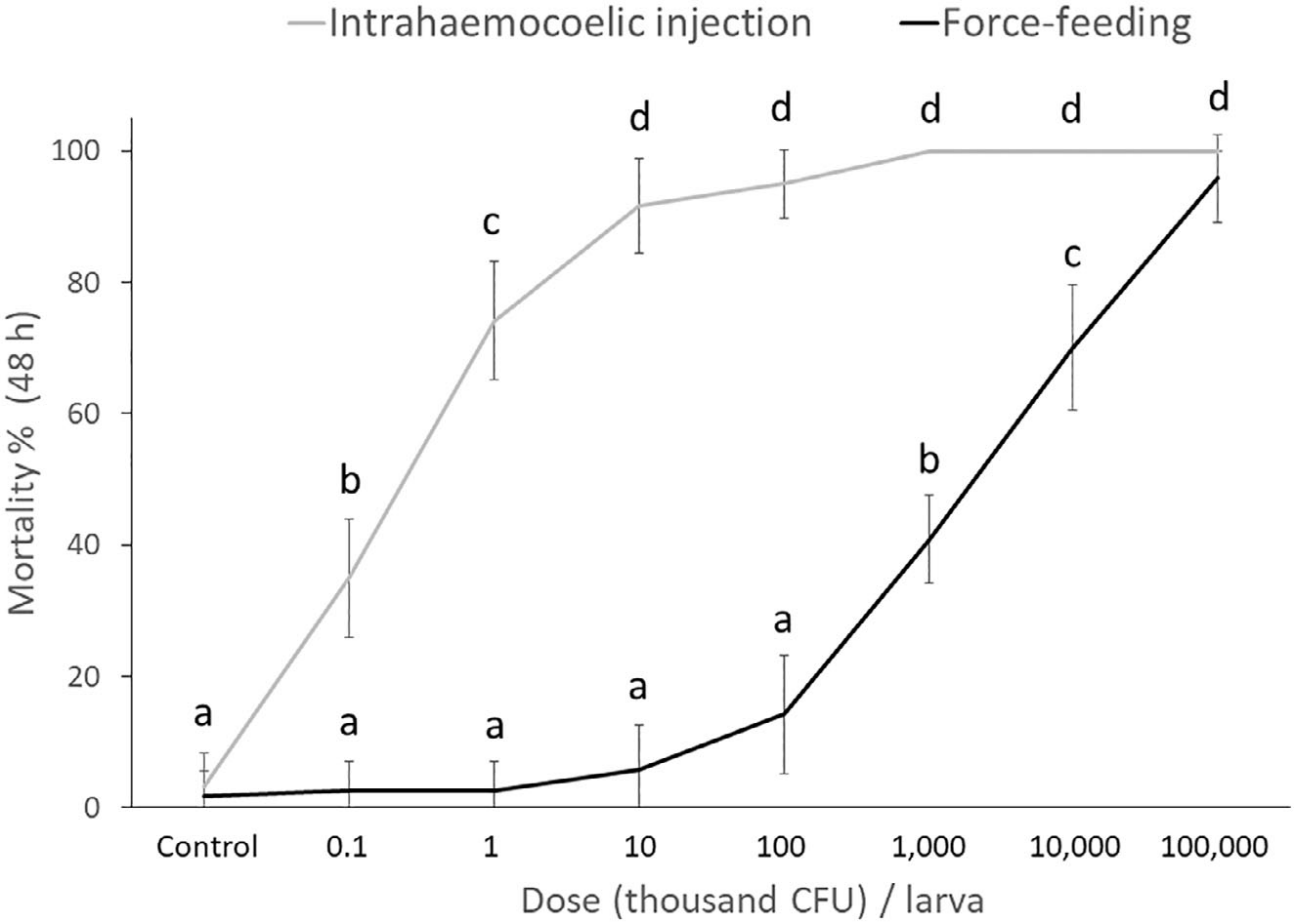
Mortality (mean ± SD) of *G. mellonella* larvae exposed to different doses of *P. protegens* cells by injection or ingestion. Different letters indicate significantly different means (two‐way ANOVA, followed by LSD test, *P* < 0.001).

The lethal effects caused by *P. protegens* strain CO1 on *G. mellonella* larvae were dose‐dependent both when administered by injection and by ingestion through force‐feeding (Fig. 3). The average percentage mortality determined 48 h after administration exceeded 90% at a dose of 10^4^ CFU per larva in the case of injection, and of 10^8^ CFU per larva in the case of ingestion of bacterial cells, whereas no mortality was observed after 48 h in larvae treated with saline solution. Mortality was significantly affected by treatment (*F*_1,176_ = 2557.21, *P* < 0.0001), dose (*F*_7,176_ = 637.32, *P* < 0.0001), and the interaction between these factors (*F*_7,176_ = 170.01, *P* < 0.0001).

According to Probit analysis, *G. mellonella* larvae appeared to be clearly more susceptible to the pathogenic action of *P. protegens* by injection than by ingestion. The LC_50_ (CI) values were 651.0 (511.0–755.0) CFU mL^–1^ by injection (slope = 1.55 ± 0.28; *χ*2 = 13.33; df = 94) and 4.0 (2.9–5.2) × 10^7^ CFU mL^–1^ by force‐feeding (slope = 1.77 ± 0.24; *χ*2 = 21.62; df = 94).

### 3.4 Effects on different target insect species

The administration of *P. protegens* strain CO1 by injection or feeding caused significant lethal effects against all target species (Table 2). On the one hand, intrahaemocoelic injection caused a rapid septicaemia in treated insects, compared to the control treated with saline, and a consequent high mortality of >80–90% (*H. armigera*: *t* = −42.51; df = 11; *P* < 0.001; *L. dispar*: *t* = −36.65; df = 11; *P* < 0.001; *M. domestica*: *t* = −34.31; df = 11; *P* < 0.001; *C. capitata*: *t* = −22.09; df = 11; *P* < 0.001). On the other, feeding caused highly significant lethal effects (>70%) on the two lepidopteran species (*H. armigera*: *t* = −25.98; df = 11; *P* < 0.001; *L. dispar*: *t* = −30.94; df = 11; *P* < 0.001), a moderate effect (58.3%) on *M. domestica* (*t* = −20.63; df = 11; *P* < 0.001) and just a slight effect (21.7%) on *C. capitata* (*t* = −8.37; df = 11; *P* < 0.001).

**Table 2:** Mortality (mean ± SE) of different insect larval species exposed to *P. protegens* by injection or feeding, after 72 h

Species	Method of exposure	Treatment	Mortality^*^%
*Helicoverpa armigera*	Injection	Treated	96.7 ± 1.4 a
		Control	4.2 ± 1.9 b
	Feeding	Treated	76.7 ± 2.8 a
		Control	1.7 ± 1.1 b
*Lymantria dispar*	Injection	Treated	97.5 ± 1.3 a
		Control	9.2 ± 1.9 b
	Feeding	Treated	71.7 ± 2.1 a
		Control	0.8 ± 0.8 b
*Musca domestica*	Injection	Treated	92.5 ± 2.8 a
		Control	3.3 ± 1.4 b
	Feeding	Treated	58.3 ± 2.7 a
		Control	0.8 ± 0.8 b
*Ceratitis capitata*	Injection	Treated	83.3 ± 2.6 a
		Control	5.8 ± 2.3 b
	Feeding	Treated	21.7 ± 2.1 a
		Control	2.5 ± 1.3 b

^*^ Different letters within each insect species and method of exposure indicate significantly different means (Student's *t*‐test, *P* < 0.001).

## 4 DISCUSSION

*Pseudomonas protegens* strain CO1, isolated primarily as a major agent of septicaemia in larvae of the wax moth, *G. mellonella*, infected with 3rd stage infective juveniles of a soil‐dwelling isolate of *S. feltiae*,^9^ was found to be significantly associated with this nematode. Such a relationship appeared to be robust, as supported by the direct and repeated isolation of the bacterium from both infected insect larvae and surface‐sterilized nematodes from different generations of infections. This result aligns with the finding of diverse bacteria in the body of *Steinernema* entomopathogenic nematodes, occasionally reported to be hosted in the intercellular space under the third‐stage cuticle.^5^ Although this resident bacterial community has remained in the background, and the core symbionts such as *X. nematophila* were considered to be the sole bacteria really characterizing EPN entomopathogenicity, it has been demonstrated more recently that other species are sustainably associated with *Steinernema* IJs.^6^ According to this study, a specific bacterial community including the genera *Brevundimonas, Ochrobactrum, Pseudochrobactrum, Achromobacter, Alcaligenes, Stenotrophomonas, Xenorhabdus* and *Pseudomonas* was found to be frequently associated with *S. carpocapsae* freshly isolated from soil, and a remarkable entomopathogenic potential of *Pseudomonas chlororaphis* and *P. protegens* isolated from nematodes was highlighted. This finding supported not only a possible involvement of these bacterial species in the entomopathogenic process caused by IJs, but also the ability of these bacteria to successfully leverage their antibiotic potential against other species, including *Xenorhabdus* spp., within the competitive insect haemocoel environment.^21^ Consistently, we found *P. protegens* strain CO1 to be the dominant bacterial species in the haemolymph of *G. mellonella* larvae inoculated with *S. feltiae*. The intrahaemocoelic injection of even a small dose (≈100 CFU per larva) resulted in a rapid increase in bacterial load in the haemolymph and equally rapid death, which is evidence of excellent adaptation of *P. protegens* to the haemocoelic environment.^7^ The ability of *P. protegens* to develop rapidly in the insect haemolymph is associated with the release of antimicrobial compounds, esoenzymes such as chitinases, and the FitD it typically produces.^22^

The expression of some of these insect virulence factors was recently observed also at the gut level in muscoid fly larvae fed with cells of strain CO1,^12^ which aligns with the pathogenic effect on *G. mellonella* larvae we observed after force‐feeding. However, the slower effectiveness of *P. protegens* after ingestion compared to injection is a clear consequence of the need to overcome the intestinal barrier, which is a natural obstacle to microorganisms to reach a suitable environment for their proliferation, namely the haemolymph.^23^ This soil‐dwelling bacterium evolved establishing a wide variety of interactions with living organisms that led to development of the specific ability to colonize plant roots stimulating their growth, to compete with phytopathogens and to act against them through antibiosis mechanisms.^24^ The expression of its entomopathogenic potential would, however, depend on the adventitious ingestion of a sufficient amount of bacterial cells by susceptible insects, and on their ability to cross the intestinal barrier. Support in this work could come from entomoparasitic nematodes, which would then be vectors for the bacterium to the insect haemocoel. The robustness of the *S. feltiae*–*P. protegens* relationship that we observed, therefore would appear to be the result of a co‐evolutionary process rather than an occasional finding, as supported by phylogenetic analyses on the microbial community of FAM species.^6^

Interestingly, the Fit complex typical of *P. protegens* shows similarity with the Makes caterpillars floppy (Mcf) toxin of the well‐known entomopathogenic nematode symbionts *Photorhabdus* and *Xenorhabdus*, which would support the sharing of a common ancestor or the possible exchange of genetic material during evolution in the same environment.^25^ According to a comparative evolutionary analysis, several transposable elements were found in *fit/mcf* genes, supporting the hypothesis of horizontal transfer during their evolution.^8^ Such observations suggest that plant‐colonizing pseudomonads may have acquired and evolved gene virulence clusters to adapt to a new ecological niche, namely the insect body, shared with other bacteria such as *Photorhabdus* and *Xenorhabdus*.^26^

Beyond the possibility of using a nematode as a carrier towards the haemocoel of the host, *P. protegens* has evolved its own potential as a stand‐alone entomopathogen.^27^ Accordingly, in our experiments on different lepidopterans and dipterans, a significant variability in the lethal effects caused by strain CO1 on different hosts was observed, which outlines a specific profile of this microorganism and adaptations that make it a promising biological control agent against certain targets. However, with respect to other well‐known insect pathogenic bacteria such as *Bacillus thuringiensis*,^28^ the establishment of a stable association with entomoparasitic nematodes makes *P. protegens* potentially more efficient in gaining access to target larvae in the soil. It remains to be understood how intimate this association of the bacterium with the nematode is. Indeed, whereas the core symbionts *Photorhabdus* and *Xenorhabdus* typically are housed in the intestine of the host nematode, the frequently associated proteobacteria were observed to be housed instead under the 3rd stage cuticle of IJs.^5^ Although it should first be proven, we cannot exclude that nematodes emerging from the body of a host insect in which a *P. protegens* septicaemia is in progress, may acquire and carry this bacterium in the intestine to a new host. This mechanism would not be in contrast to the well‐known role of the core symbionts in initiating infections, whereas *P. protegens*, when present, might leverage its arsenal of antibiotic substances to the detriment of other bacterial species normally involved in septicaemia.^29^

Further experiments, especially under field conditions, are needed to determine the extent to which this association with *Steinernema* may constitute a competitive advantage for an emerging entomopathogenic bacterium whose behaviour in the environment is still poorly known.

## CONFLICT OF INTEREST DECLARATION

The authors declare that there is no conflict of interest.

## Data Availability

The data that support the findings of this study are available upon reasonable request.
